# Virtual experiments in computational magnetism with mag2exp

**DOI:** 10.1038/s41524-025-01686-3

**Published:** 2025-07-01

**Authors:** Samuel J. R. Holt, Martin Lang, James C. Loudon, Thomas J. Hicken, Dieter Suess, David Cortés-Ortuño, Swapneel A. Pathak, Marijan Beg, Kauser Zulfiqar, Hans Fangohr

**Affiliations:** 1 Max Planck Institute for the Structure and Dynamics of Matter, Hamburg, Germany; 2 Center for Free-Electron Laser Science, Hamburg, Germany; 3 Department of Materials Science and Metallurgy, University of Cambridge, Cambridge, UK; 4 PSI Center for Neutron and Muon Sciences, 5232 Villigen PSI, Switzerland; 5 Faculty of Physics, University of Vienna, Vienna, Austria; 6 Research Platform MMM Mathematics-Magnetism-Materials, University of Vienna, Vienna, Austria; 7 Departamento de Física, Universidad Técnica Federico Santa María, Valparaíso, Chile; 8 Department of Earth Science and Engineering, Imperial College London, London, UK; 9 Department of Physics, University of Hamburg, Hamburg, Germany; 10 Faculty of Engineering and Physical Sciences, University of Southampton, Southampton, UK

**Keywords:** Condensed-matter physics, Ferromagnetism, Computational methods, Imaging techniques, Microscopy

## Abstract

We have designed and implemented the Python package mag2exp, which enables researchers to perform a range of virtual experiments given a spatially resolved vector field for the magnetization, a typical result from computational methods to simulate magnetism such as micromagnetics. This software allows experimental measurements such as magnetometry, microscopy, and reciprocal space based techniques to be simulated in order to obtain observables that are comparable to those of the corresponding experimental measurement. Such virtual experiments tend to be more economic to carry out than actual experiments. There are many uses for virtual experiments, including (i) choosing the best experimental techniques and assessing their feasibility prior to experimentation, (ii) fine tuning experimental setup, (iii) guiding the experiment by conducting concurrent simulations of the measurement, and (iv) interpreting the experimental data at a later point though both qualitative and quantitative methods.

## Introduction

There are many experimental techniques that allow magnetic properties to be probed, but often they cannot unambiguously identify the magnetic structure. Complementing experimental results with insights from theoretical and computational studies can give a fuller and more complete picture of the physics of a system.

Computational experiments—such as the use of computer simulation to study the behavior of a physical system under certain model assumptions—are often more economical, easier to set up and carry out than their real-world counterparts, and allow researchers to probe quantities that are unable to be measured in experiments. We can regard the use of these computer simulations as *virtual experiments*.

Virtual experiments, while by definition being an approximation of reality, can have a variety of applications before, during, and after real-world experiments. This includes enabling researchers to run exploratory computational experiments to examine what techniques might be the most sensitive to a given phenomenon, and provide justification for difficult and expensive experiments. Virtual experiments can be used concurrently with real-world experiments to inform an experiment and guide the research process. Virtual experiments can aid the processing of experimental data by providing additional insights into the measured data and enabling a deeper understanding of the system being studied.

In the context of magnetism research, computer simulations are often realized through micromagnetic simulations in which the temporal and spatial behavior of the magnetization vector field can be computed. Assuming the model assumptions are appropriate, this can reveal the underlying physics and help to complement experimental work as outlined above. However, in the experiments we often cannot directly access this magnetization vector field and instead one can only observe derived properties, such as a spatially averaged magnetic moment as measured in x-ray holography or an accumulated electron phase difference as measured in Lorentz transmission electron microscopy (LTEM). The direct comparison of the simulations (i.e., the magnetization vector field) with these experimentally accessible observables is often non-trivial and this hinders the interpretation of the data.

In this work, we address this problem by implementing algorithms in order to perform virtual experiments which map a magnetization vector field to observables that would be obtained from real experiments. The outcomes of these virtual experiments can then be compared directly with the outcomes of the experiments.

A few tools that address one experimental technique at the time have been published^1,2^. Here, we provide algorithms to simulate multiple experimental techniques in a more systematic and re-usable way to benefit the wider community. The software, published together with the manuscript, is an essential part of the work.

In this paper, we present the Python package mag2exp (https://github.com/ubermag/mag2exp, https://ubermag.github.io) that enables a range of virtual experiments to be performed on arbitrary magnetic structures. The mag2exp package is integrated in the micromagnetic simulation environment Ubermag^3–5^. The input can be any arbitrary magnetization field on a finite-difference mesh such as those produced using micromagnetic.

**Fig. 1:**
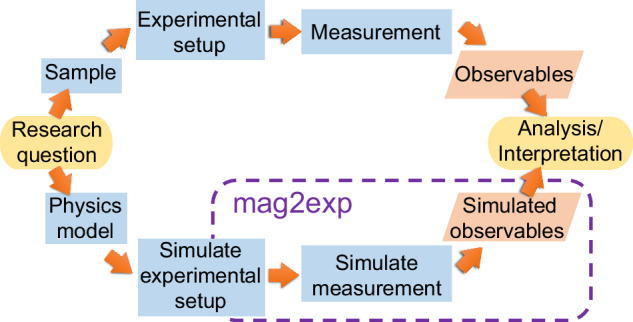
Research workflows. Representative experimental (top) and computational (bottom) research workflows highlighting the role of mag2exp and the parallels between the two workflows.

Figure 1 shows the role of mag2exp in an example research workflow. The top path of the figure represents an approximate experimental workflow that leads from a research question, to the choice of a sample with the appropriate physics, to the set up of a relevant experiment, to the measurement of the desired observable which is to be used for analysis and interpretation.

The bottom path in Fig. 1 shows an approximate computational workflow which mirrors the experimental workflow. Firstly a physics model is set up based on the system under investigation: it needs to reflect the essential physics of the sample. For example this could be a micromagnetic model. The system is then modified to reflect the relevant conditions of the experimental setup. This typically involves the execution of a micromagnetic simulation to create a (often spatially and temporally resolved) magnetization vector field that approximates the magnetization in the real sample. The mag2exp package can then *simulate the measurement*—for example computing the MFM image that would be observed and provide the (simulated version) of the observables. In the last “Analysis/Interpretation” step, the observables from experimental measurement (top path) and observables based on simulation of the physics and the simulated measurement (bottom path) can be compared, analyzed, and interpreted together based on the researchers’ needs. This could include, but is not limited to: qualitative and quantitative comparisons, feasibility studies, refinements of analytical magnetization models using experimental results.

The mag2exp package aims to help bridge the gap between computational magnetism simulations and experiments, leading to the potential for simulations to inform real-world experiments and vice versa. There is also further scope for potential applications of mag2exp such as the generating artificial data sets that can be used in a machine learning context.

In the following, we use micromagnetic modeling in combination with mag2exp as an example workflow. Details of the methodology, the mag2exp package and the micromagnetic model we use are given in Section “Methods”. In section “Results” we discuss simulation of measurements for a select number of experimental techniques that mag2exp currently supports. These are grouped into Magnetometry (Section “DC magnetometry” and “Torque magnetometry”), Microscopy (Section “Lorentz transmission electron microscopy”, “x-ray holographic microscopy”, and “Magnetic force microscopy”), Reciprocal space (Section “Small-angle neutron scattering”), and Dynamic measurements (Section “Ferromagnetic resonance”). Finally, we close with a discussion in Section “Discussion”. Supplementary material is provided to enable reproduction of all figures presented and includes further examples illustrating each experimental technique, as well as extensions such as tilt-series measurements and inverse problems^6^.

## Results

### DC magnetometry

Magnetometry techniques are a widely used and important tool for characterizing many materials. These techniques are usually bulk sensitive and used to examine properties of magnetization in the sample such as its saturation magnetization, the spatially averaged magnetization direction, and magnetic torque.

DC magnetometry is a measurement technique that probes the magnetization of a sample in an externally applied magnetic field. The process of calculating the overall magnetization of a sample from micromagnetic simulations is to take the average magnetization of the sample. For the DC magnetometry technique in mag2exp, the technique reference frame is defined to be the same as the sample reference frame. In mag2exp, the magnetization is calculated only over the valid volume *V*

1 $${{\bf{M}}}_{{\rm{ave}}}=\frac{{\int}_{V}{\bf{M}}({\bf{r}}){{\rm{d}}}^{3}r}{{\int}_{V}{{\rm{d}}}^{3}r}\in {{\mathbb{R}}}^{3}$$

$$V=\{{\bf{r}}\,| \,{\bf{r}}\in {{\mathbb{R}}}^{3}\,\text{and}\,{\rm{valid}}({\bf{r}})={\rm{True}}\}.$$

The use of this valid property allows highly complex heterogeneous systems to be measured, giving realistic ensemble averages. Using an input of a spatially resolved magnetization field **M**(**r**) with name field, the spatially averaged magnetization **M**_ave_ can be calculated using


1 mag2exp.magnetometry.magnetization(field)


which returns a single DC magnetization vector in A/m. DC magnetometry measurements are commonly carried out experimentally and the data interpretation is often straightforward. However, in general it can be difficult to account for effects such as demagnetization and interpret data for ensemble systems. This leads to an advantage of performing computational micromagnetics to assist in data interpretation for complex geometries, where one can simulate a computational volume matching the shape of the experimental sample.

**Fig. 2:**
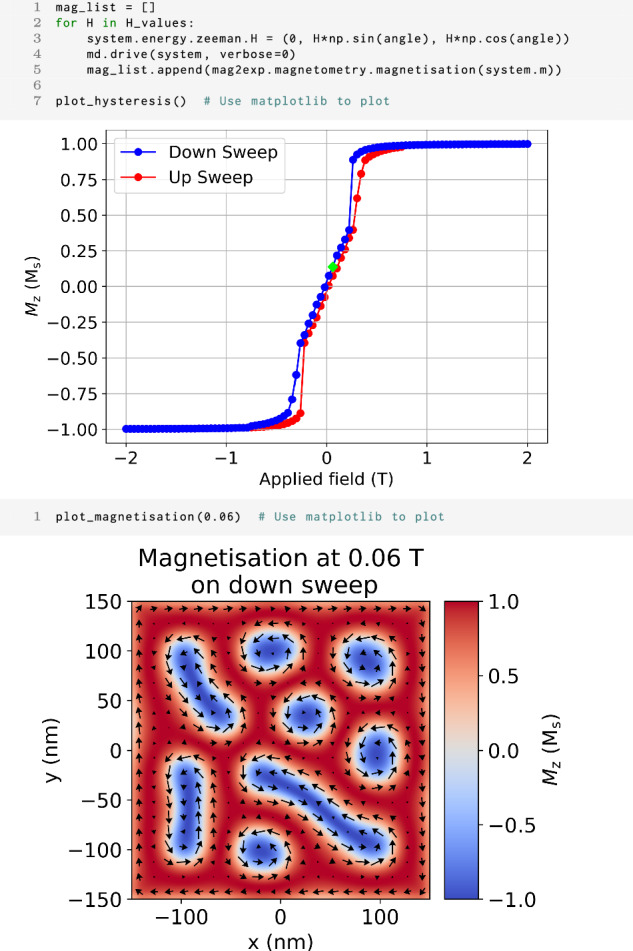
DC magnetometry. The first cell shows the code to produce a hysteresis curve using micromagnetic simulations and mag2exp with the applied field along the *z* direction. Only the *z* component of the magnetization is plotted for the down (blue) and up (red) sweep. The second cell shows the magnetization structure of the *z* = 0 nm plane of the sample in the down sweep of the hysteresis curve at 0.06 T. The arrows show the direction of in-plane magnetization while the color indicates the *z* component of the normalized magnetization. The green diamond in the first plot shows data used for the 0.06 T magnetization plot in the second cell.

As mag2exp is a Python package and integrated in the Ubermag framework, it is straightforward to recreate hysteresis loops that would be performed experimentally. Figure 2 shows the hysteresis curve of the micromagnetic simulation by varying the magnitude of the applied magnetic field in the *z* direction. The simulated measurement shows that at fields over 1 T the sample is saturated, however, there is hysteretic behavior of the down and up sweeps of the field. This hysteresis loop has similar characteristic to those of FeGe^7^, however, it differs due to the use of a zero-temperature model in micromagnetics. One of the benefits of performing these hysteresis loops using micromagnetic simulation is that the microscopic magnetization at specific applied fields can be probed to look at the mechanism of this hysteresis as seen in Fig. 2 (bottom plot).

### Torque magnetometry

Torque magnetometry is a technique where a sample is placed in an external magnetic field and the torque on the sample due to the externally applied field is measured. This technique is commonly used to measure the anisotropy of materials by applying a large enough magnetic field to saturate the sample and then rotating the sample relative to the field to obtain the easy and hard axes. However, similarly to other bulk techniques such as DC magnetometry, it can be difficult to interpret the experimental results of torque magnetometry in ensemble systems and also due to effects such as demagnetization. It is also hard to predict the torque magnetometry results for magnetic states that are not in the saturated state. mag2exp opens another avenue for interpretation of results by computationally simulating the torque of a magnetic state.

Magnetic torque ***τ*** arises from the cross product of the magnetic moment **m** and the magnetic flux density **B,**

2 $${\boldsymbol{\tau }}={\bf{m}}\times {\bf{B}}$$

3 $$={\mu }_{0}{\bf{m}}\times {{\bf{H}}}_{{\rm{app}}},$$

where **H**_app_ is the applied magnetic field^8^. The total torque of the system can be expressed in terms of the magnetization **M**

4 $${\boldsymbol{\tau }}=\frac{{\mu }_{0}{\int}_{V}{\bf{M}}({\bf{r}})\times {{\bf{H}}}_{{\rm{app}}}\,{\rm{d}}V}{{\int}_{V}{\rm{d}}V}\in {{\mathbb{R}}}^{3},$$

$$V=\{{\bf{r}}\,| \,{\bf{r}}\in {{\mathbb{R}}}^{3}\,\text{and}\,{\rm{valid}}({\bf{r}})={\rm{True}}\}.$$

Similarly to DC magnetometry, us of the valid property allows highly complex heterogeneous systems to be measured, giving realistic ensemble values. For the torque magnetometry technique in mag2exp, the technique reference frame is defined to be the same as the sample reference frame. The torque is calculated using


1 mag2exp.magnetometry.torque(field, ...)


The magnetization field is passed to the function along with the applied magnetic field.

**Fig. 3:**
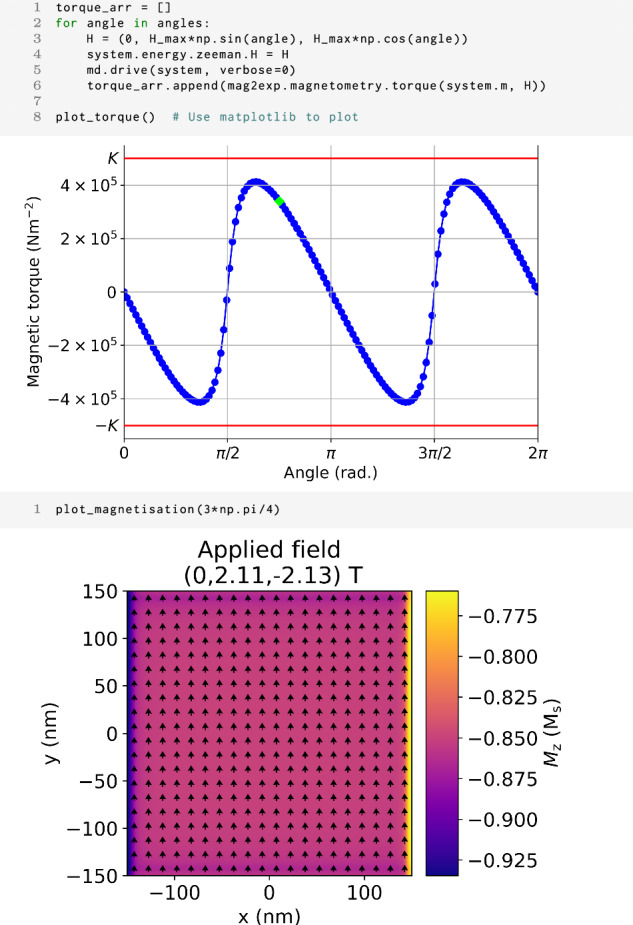
Torque magnetometry. The first cell shows the code to produce a torque magnetization curve using micromagnetic simulations and mag2exp with an applied field of 3 T rotating in the yz plane. The angle denotes the angle from the *z* axis in the *yz* plane. The red lines indicate the uniaxial anisotropy constant *K* = 5 × 10^5^ Jm^−3^. The second cell shows the magnetization structure of the *z* = 0 nm plane of the sample in the down sweep of the torque curve with an applied field of (0, 2.11, −2.13) T. The arrows show the direction of in-plane magnetization while the color indicates the *z* component of the normalized magnetization in units of the saturation magnetization. The green diamond in the first plot shows data used for the (0, 2.11, −2.13) T magnetization plot in the second cell.

Using the example of the micromagnetic model outlined in Section “Methods”, with the anisotropy constant *K* increased to 5 × 10^5^ Jm^−3^ to enhance the effects, a magnetic field of 3 T can be applied along the *z* axis to attempt to saturate the magnetic state. The applied field is then rotated in the *yz* plane and the torque is calculated. Figure 3 shows a torque magnetization curve calculated from micromagnetic simulations. The data shows a characteristic sheared sinusoidal wave with a period of *π* as expected for uniaxial anisotropy. The sine wave peaks at a value close of the uniaxial anisotropy constant of *K* = 5 × 10^5^ Jm^−3^ as theoretically expected^9^. Deviations from a perfect sinusoid, notably the observed amplitude variations and shearing, originate primarily from demagnetization effects linked to the sample geometry. Experimentally, in the absence of detailed micromagnetic calculations, the standard equations used to extract anisotropy constants from torque magnetometry data typically assume that the applied external field is sufficiently strong to fully align the magnetization along the applied field direction^10^. In practice, this is generally not the case. As shown for our cuboidal geometry with approximate demagnetization factors of *N*_*z*_ = *N*_*y*_ ≈ 0.077 and *N*_*z*_ ≈ 0.909, even in an applied field of 3 T significant deviations occur due to demagnetization effects^11^. These are challenging to accurately account for in experimental torque magnetometry, often leading to systematic errors in derived parameters like anisotropy constants. However, using a tool like mag2exp, one can compute the spatially varying magnetization vector field, the corresponding spatially resolved demagnetization field, thereby overcoming typical experimental limitations.

### Lorentz transmission electron microscopy

Microscopy techniques enable the imaging of magnetic structures on a nanometer length scale. This makes them powerful techniques to corroborate micromagnetic simulations due to the similarity of the length scales probed. Microscopy techniques have been vital in the discovery of magnetic structures and investigations into their properties^12–15^.

LTEM^16^ is a popular method for imaging magnetic structures in thin lamellae^12–14^. However, given a 3-dimensional magnetic structure, it is not always easy to predict what images produced using Lorentz TEM will look like. This can lead to the misidentification of magnetic structures, such as type-2 magnetic bubbles being identified as biskyrmions^17^. To assist accurate identification, mag2exp has been developed to help simulate Lorentz TEM techniques.

In a transmission electron microscope, a beam of electrons, each with a kinetic energy typically hundreds of keV, travels down the microscope column and passes through a thin (typically 100 nm) sample called a lamella. In mag2exp, the technique reference frame is defined with the electrons traveling in the *z* direction. If the sample is magnetic, the electrons are deflected by the Lorentz force **F** = −*e***v** × **B** as they pass through where *e* is the electron charge and **v** is the electron velocity. This means that electron microscopy is sensitive to the magnetic flux density **B** from the sample rather than the magnetization **M** and that due to the nature of the cross-product, it is sensitive only to the component of **B** normal to the electron beam.

The effect of the beam deflection is that the electrons emerge from the sample having acquired a phase shift given by Aharonov-Bohm equation^18^:

5 $${\phi }_{{\rm{m}}}(x,y)=-\frac{2\pi e}{h}\mathop{\int}\nolimits_{-\infty }^{\infty }{A}_{z}(x,y,z)\,{\rm{d}}z,$$

where *e* is the electron charge, *h* is Planck’s constant, and *A*_*z*_ is the *z* component of the magnetic vector potential.

Beleggia and Zhu^19^ showed that if the Fourier transform of this phase shift is taken, it can be written in terms of the magnetization as

6 $${\widetilde{\phi }}_{m}({k}_{x},{k}_{y})=\frac{ie{\mu }_{0}{k}_{\perp }^{2}}{h}\frac{{\left[{\widetilde{{\bf{M}}}}_{I}({k}_{x},{k}_{y})\times {{\bf{k}}}_{\perp }\right]}_{z}}{{\left({k}_{\perp }^{2}+{k}_{{\rm{c}}}^{2}\right)}^{2}},$$

where ${\widetilde{{\bf{M}}}}_{I}({k}_{x},{k}_{y})$ is the Fourier transform of the magnetization integrated along the incident beam direction, *μ*_0_ is the permeability of free space, **k**_⊥_ represents the in-plane components of the wave vector **k**, and *k*_c_ is the Tikhonov filter which prevents a singularity when ${k}_{\perp }^{2}=0$^20^.

After the electron beam exits the sample, electromagnetic lenses focus it and create an image. The electron wavefunction for an in-focus image corresponds to the wavefunction the electron beam had as it exited the specimen and the magnetic contribution to this wavefunction is ${\psi }_{0}=\exp (i{\phi }_{m})$. It can be seen that the intensity of an in-focus image will not contain any magnetic features as *I*_0_ = ∣*ψ*_0_∣^2^ = 1.

The are several methods to acquire images that are sensitive to magnetism. The most direct method is off-axis electron holography which is an interferometric technique from which the phase can be recovered directly^21^. In mag2exp the phase can be calculated from the magnetization field in real space using


1 mag2exp.ltem.phase(field, ...)


Where the relevant parameters can be defined such as the size of the Tikhonov filter. This can be compared directly with the phase recovered by electron holography.

Another method is to acquire out-of-focus images rather than in-focus images^16^. The technique is also called Fresnel imaging^22^. It is usually a more convenient experiment than off-axis holography but the images obtained are harder to interpret.

The wavefunction corresponding to an out-of-focus image, *ψ*_Δ*f*_, can be calculated by taking the in-focus wavefunction *ψ*_0_ and propagating it through free space by a distance Δ*f* known as the defocus. This operation is performed most conveniently in Fourier space^22,23^ via

7 $${\widetilde{\psi }}_{\Delta f}({k}_{x},{k}_{y})={\widetilde{\psi }}_{0}({k}_{x},{k}_{y}){e}^{-i\chi (k)},$$

where

8 $$\begin{array}{lll}\chi (k)\;=\,-\pi \Delta f\lambda {k}^{2}\\\qquad\quad\;\;\;+\,\frac{1}{2}\pi {C}_{{\rm{s}}}{\lambda }^{3}{k}^{4}\\\qquad\quad\;\;\;+\,\pi {C}_{{\rm{a}}2}\lambda {k}^{2}\sin [2(\phi -{\phi }_{a})].\end{array}$$

The first term is known as the defocus term. The second term originates from the spherical aberration of the imaging lens and is characterized by the coefficient of spherical aberration, *C*_s_. The third term corresponds to the two-fold astigmatism of the lens, where *C*_a2_ is the two-fold astigmatism coefficient, *ϕ* is the azimuthal angle, and *ϕ*_*a*_ is the azimuthal orientation of the aberration. The intensity of the defocused image is then given by

9 $${I}_{\Delta f}(x,y)=| {\psi }_{\Delta f}(x,y){| }^{2}.$$

The defocused image can be calculated in mag2exp from the phase in real space using


1 mag2exp.ltem.defocus_image(phase, ...)


The function takes additional parameters such as the accelerating voltage of the electron beam (or the wavelength of the electrons) and the defocus. The output of this function can be compared directly with defocused images obtained experimentally.

Other useful quantities such as the integrated magnetic flux density can also be calculated using the mag2exp software package.

**Fig. 4:**
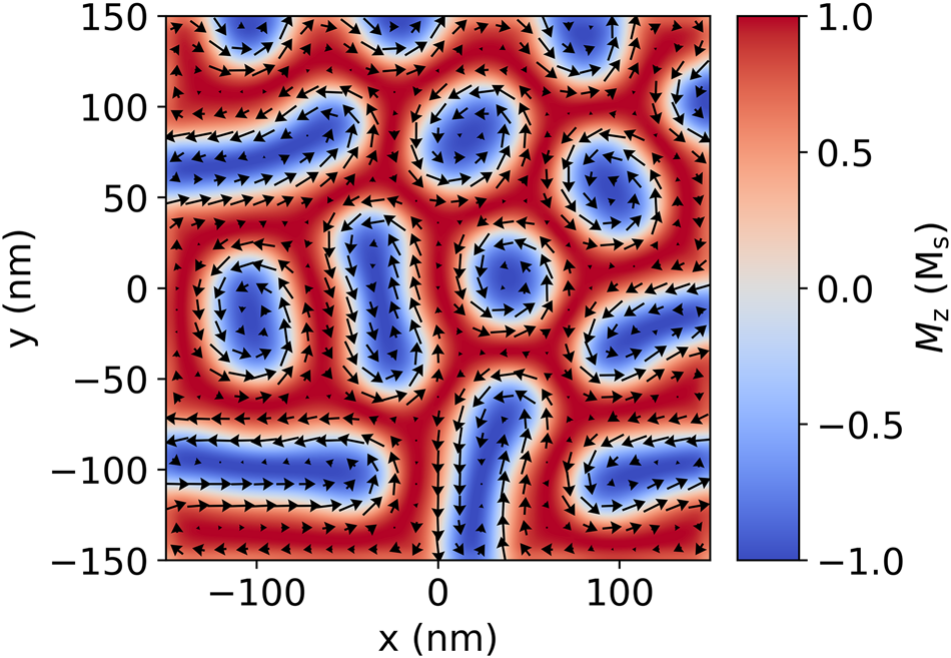
Magnetization structure. Center plane of the magnetization structure produced using energy minimization for a randomized starting configuration for the energy terms detailed in Section “Methods” under an applied magnetic field of 0.1 T.

**Fig. 5:**
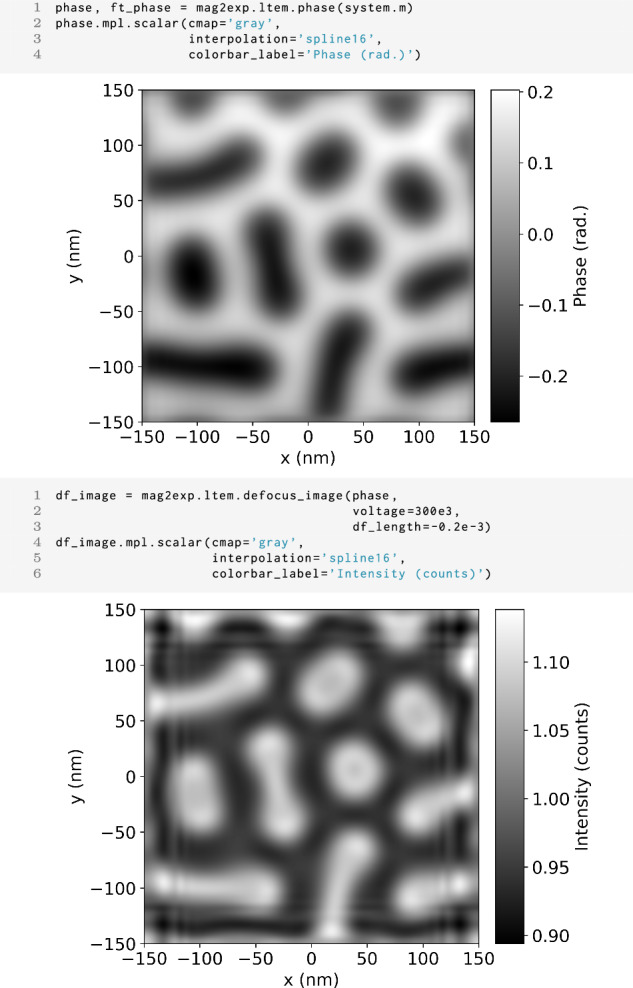
Lorentz TEM. The first cell shows the code to simulate and plot the phase of the electron beam for Lorentz TEM of the magnetic structure shown in Fig. 4. The second cell shows the code to simulate and plot a defocused image with an accelerating electron voltage of 300 kV and a defocus of −0.2 mm.

As an example, Fig. 4 shows the magnetization produced by the micromagnetic model detailed in Section “Methods” following energy minimization for a randomized starting configuration under an applied magnetic field of 0.1 T. Using this magnetic structure, Fig. 5 shows the phase and an image which was simulated with an accelerating electron voltage of 300 kV and a defocus of −0.2 mm. These represent typical experimental parameters used in Lorentz TEM. In the defocused image, the Bloch magnetization texture appears as areas of high intensity in the center of the skyrmion-like objects, matching well what is seen for these kinds of magnetic textures in literature including for FeGe^12,24^. Fringes can also be seen in the defocused image caused by the finite nature of the geometry.

The combination of the ability of mag2exp to simulate LTEM patterns and also rotate magnetic structures using the FieldRotator enable very powerful experiments such as tilt series to be carried out.

There are a number of other software packages capable of simulating Lorentz TEM techniques such as *PyLorentz*^2^ which allows the simulations of the phase and defocused images and also reconstructions of the magnetic flux density from Lorentz TEM images. Results of mag2exp were validated with scripts written for defocused imaging in the Semper image processing language^25^.

### x-ray holographic microscopy

x-ray holography is a technique which uses circularly polarized light to take advantage of the magnetic circular dichroism effect: left and right circularly polarized light are absorbed differently in the presence of a magnetic field parallel to the propagation direction of the light. Hence, this effect is only sensitive to the magnetization parallel to the propagation direction of the light. The wavelengths of the x-rays can be tuned to the absorption edges of the different elements present in the sample allowing for element-specific magnetization to be measured. However, when calculating x-ray holography images in mag2exp we use the approximation that the magnetization is not element specific and therefore give the output in terms of integrated magnetization rather than scaling by scattering factors to give an intensity^26,27^.

In x-ray holography, a coherent beam of circularly polarized x-rays is incident on the sample and a reference slit. The scattered x-rays interfere and form a hologram which is recorded by the detector. The measured hologram can subsequently be combined with a differential filter and Fourier transformed to reconstruct the holographic image which contains information on the out-of-plane magnetization of the sample.

For the x-ray holography technique in mag2exp, the technique reference frame is defined with the incident beam propagating along the *z* direction. From a magnetization field, the reconstructed holographic image can be calculated from the integral of the magnetization along the path of the beam^26,27^

10 $$I(x,y)=\int{M}_{z}(x,y,z)\,{\rm{d}}z.$$

This can be obtained from the magnetization field using the function


1 mag2exp.x_ray.holography(field, ...)


To obtain more experimentally realistic images, automatic convolution with a Gaussian can be used in order to simulate the finite resolution of the instrument. Similarly to LTEM, combining the simulated x-ray holography technique with the FieldRotator enables simulations of tilt-based experiments, which has important applications to research areas such as magnetic tomography.

**Fig. 6:**
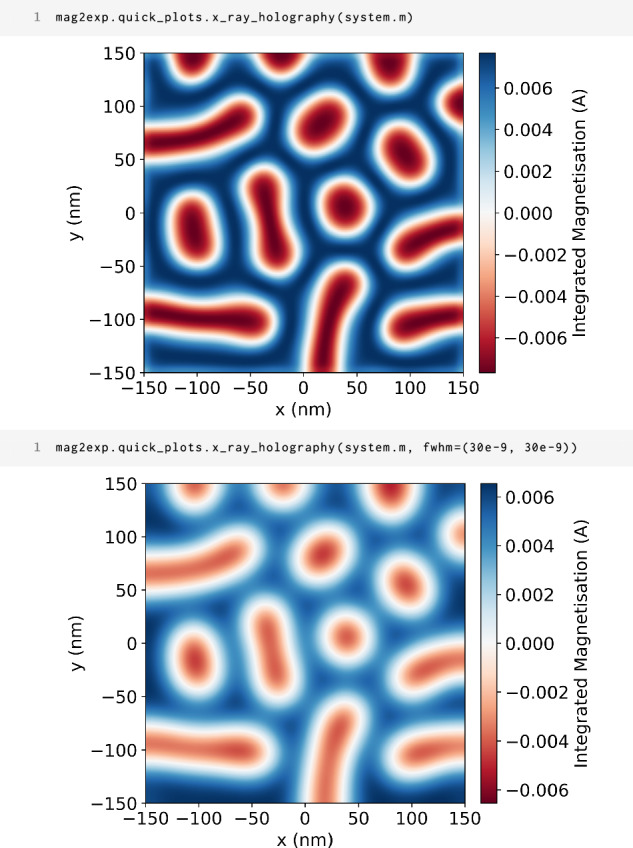
x-ray holography. The first cell shows the quick plots code to simulate x-ray holography images for the magnetic structure shown in Fig. 4 with no filter. The second cell shows the quick plots code to simulate x-ray holography images with a 20 nm Gaussian filter.

Figure 6 shows the x-ray holography images of the magnetization structure shown in Fig. 4 with and without the application of a Gaussian filter. As expected, when a 30 nm full width at half maximum Gaussian filter is applied, the features become slightly obscured and thus more similar to experimentally obtained measurements obtained on FeGe^28^. Incorporating the finite resolution into the simulation means that it is possible to investigate what magnetic features can be resolved experimentally using x-ray holography without the need for costly experiments.

### Magnetic force microscopy

Magnetic force microscopy (MFM) is a technique which relies on the magnetic interaction between an oscillating cantilever with a magnetic tip and the sample’s stray magnetic field in order to probe the sample’s magnetization texture. In MFM, in dual pass mode, the cantilever first rasters across the surface while oscillating at the cantilever’s resonant frequency, mapping the topography of the surface^29^. The same line scan is then performed again at a set lift-off height *z*_0_ from the surface of the sample. It is this second scan which is sensitive to the stray magnetic field and can be mapped through the phase shift of the resonant frequency of the cantilever. For the MFM technique in mag2exp, the technique reference frame is defined with the cantilever oscillating in the *z* direction. Due to its surface sensitivity, MFM is often used to image the magnetic structure in thin films and samples with flat surfaces.

In the technique reference frame, the phase shift of the cantilever can be expressed as

11 $$\Delta \phi =\frac{Q{\mu }_{0}}{k}\left(q\frac{\partial {H}_{{\rm{s}}z}}{\partial z}+{{\bf{M}}}_{{\rm{t}}}\cdot \frac{{\partial }^{2}{{\bf{H}}}_{{\rm{s}}}}{\partial {z}^{2}}\right),$$

where **H**_s_ is stray magnetic field, *Q* and *k* are the quality factor and spring constant of the cantilever respectively, and *q* and **M**_t_ are the effective magnetic monopole moment and magnetic dipole moment of the tip^29,30^. The effective magnetic monopole moment is sensitive to the first order derivative of the stray field in the *z* direction and the magnetic dipole moment sensitive to the second order derivative of the stray field in the *z* direction. This expression, however, describes a point like tip and hence this result can be convolved with a tip function in order to give more realistic images.

For the simulation of the measurement, we need to know the stray field **H**_s_ as a function of space in the area where the magnetic tip of the MFM is probing the stray field. The commonly employed computational methods in micromagnetism do only compute the stray field inside the sample. To get access to the stray field in the region above the sample where the tip oscillates, we use an *airbox* method.

**Fig. 7:**
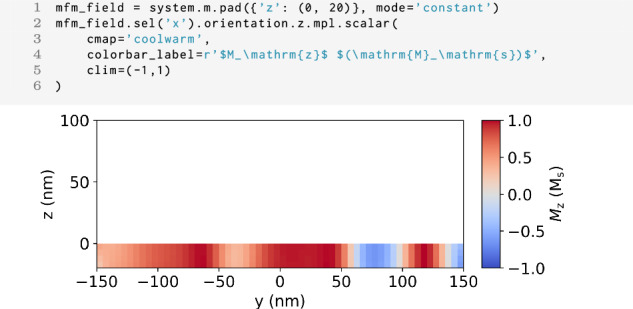
MFM airbox. Code to add 20 cells in the z direction to the magnetic structure shown in Fig. 4 and plot the magnetization structure in the *x* = 0 nm plane used for MFM calculations. The region above *z* > 0 nm is an airbox and has zero magnetization.

The airbox is the region of free space above the sample where we have to simulate the stray field in order to calculate MFM images. Figure 7 shows a *yz* cross section of the simulation region. The sample located at *z* < 0 nm has the same magnetic structure as shown in Fig. 4. Additionally, the simulation contains a region of free space with size 300 × 300 × 100 nm^3^ at *z* > 0 nm, the airbox. This allows for the calculation of the MFM response at arbitrary lift-off height within the airbox. We can choose the lift-of height in post by cutting a plane at the desired *z* position.

In mag2exp, the phase shift can be calculated using


1 mag2exp.mfm.phase_shift(field, ...)


Where parameters such as the tip’s magnetic dipole moment, the tip’s effective monopole moment, the quality factor and spring constant of the cantilever can be passed. Internally, the stray field is calculated using OOMMF^31^ and together with the experimental parameters a phase shift is calculated. This phase shift is calculated for the sample and the airbox, however, it is only physically meaningful in the airbox.

**Fig. 8:**
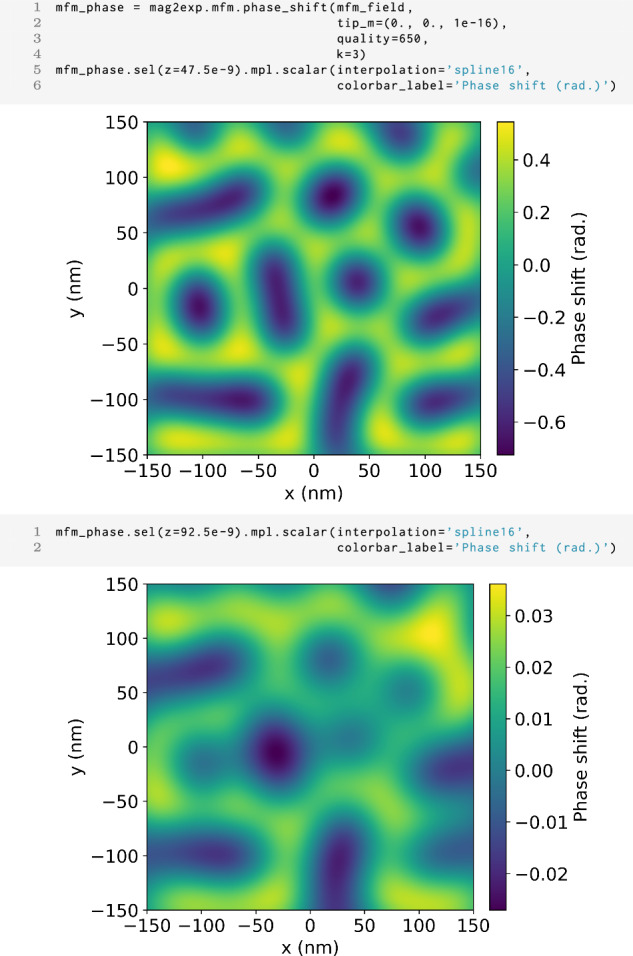
MFM images. Commands to produce the phase shift of the cantilever for the magnetic structure shown in Fig. 7 at Top: 47.5 nm and Bottom: 92.5 nm lift off heights. The phase shift has been calculated for a quality factor of 650, a spring constant of 3 Nm^−1^ and a tip with a magnetic dipole moment in the *z* direction of 1 × 10^−16^ Am^−1^.

Figure 8 shows the MFM phase shift at multiple typical lift-off heights highlighting how mag2exp can be used to investigate the best lift-off height in order to image the intended magnetic structures. These findings align well with previously reported results on FeGe^32^, underscoring the reproducible nature and accuracy of the approach.

In order to account for the finite resolution of the tip, mag2exp allows convolution with a Gaussian filter in an analogous way to that described for x-ray holography.

### Small-angle neutron scattering

Reciprocal space techniques are powerful techniques for examining the periodicity of magnetic structures. Techniques such as small-angle neutron scattering (SANS) are responsible for the discovery of magnetic textures such as skyrmions^33,34^.

In SANS a beam of neutrons is incident on a sample in a transmission geometry and the neutrons scatter as they travel through the sample due to both nuclear and magnetic forces. In mag2exp only scattering due to the magnetization of the sample is considered. The scattering pattern is related to the periodicity of the magnetic structure. The polarization of the neutrons before and after scattering can be controlled to yield more information about the magnetization structure from spin-flip cross sections^35,36^. For the SANS technique in mag2exp, the technique reference frame is defined to be the same as the sample reference frame.

SANS cross sections can be calculated with the use of the magnetic-interaction vector

12 $${\bf{Q}}=\hat{{\bf{q}}}\times \left[\widetilde{{\bf{M}}}\times \hat{{\bf{q}}}\right],$$

where $\hat{{\bf{q}}}$ is the unit scattering vector and $\widetilde{{\bf{M}}}$ is the Fourier transform of the magnetization^37^. The scattering vector is defined as

13 $${\bf{q}}={{\bf{k}}}_{{\rm{f}}}-{{\bf{k}}}_{{\rm{i}}},$$

where **k**_i_ and **k**_f_ are the incident and scattered wave vectors, respectively. The contribution to the cross sections due to magnetic scattering can be calculated using

14 $$\frac{{\rm{d}}{\mathbf{\Sigma }}}{{\rm{d}}\Omega }\propto | {\bf{Q}}\cdot {\boldsymbol{\sigma }}{| }^{\circ 2},$$

where ***σ*** is the Pauli vector and ∘ is the Hadamard power (element wise power of the matrix elements)^38^. In mag2exp the Pauli vector can be rotated to account for polarization in arbitrary directions.

The cross section takes the form of a 2 × 2 matrix with each element of the matrix representing a different spin-flip cross section

15 $$\frac{{\rm{d}}{\mathbf{\Sigma }}}{{\rm{d}}\Omega }\equiv \left(\begin{array}{rc}\frac{{\rm{d}}{\Sigma }^{++}}{{\rm{d}}\Omega }&\frac{{\rm{d}}{\Sigma }^{-+}}{{\rm{d}}\Omega }\\ \frac{{\rm{d}}{\Sigma }^{+-}}{{\rm{d}}\Omega }&\frac{{\rm{d}}{\Sigma }^{--}}{{\rm{d}}\Omega }\end{array}\right),$$

where the superscripts represent the spin flips, e.g., + − represents a positive to negative spin flip and − − represents a negative to negative spin flip. The half spin-flip cross sections can then be expressed as

16 $$\frac{{\rm{d}}{\Sigma }^{+}}{{\rm{d}}\Omega }=\frac{{\rm{d}}{\Sigma }^{++}}{{\rm{d}}\Omega }+\frac{{\rm{d}}{\Sigma }^{+-}}{{\rm{d}}\Omega },$$

17 $$\frac{{\rm{d}}{\Sigma }^{-}}{{\rm{d}}\Omega }=\frac{{\rm{d}}{\Sigma }^{--}}{{\rm{d}}\Omega }+\frac{{\rm{d}}{\Sigma }^{-+}}{{\rm{d}}\Omega },$$

while the unpolarized cross section takes the form

18 $$\frac{{\rm{d}}\Sigma }{{\rm{d}}\Omega }=\frac{1}{2}\left(\frac{{\rm{d}}{\Sigma }^{+}}{{\rm{d}}\Omega }+\frac{{\rm{d}}{\Sigma }^{-}}{{\rm{d}}\Omega }\right).$$

Once these cross sections are obtained it is possible to calculate other quantities such as the chiral function

19 $$-2\pi i\chi =\frac{{\rm{d}}{\Sigma }^{+-}}{{\rm{d}}\Omega }-\frac{{\rm{d}}{\Sigma }^{-+}}{{\rm{d}}\Omega },$$

which describes the difference between the spin flip cross sections.

In mag2exp, the cross section $\frac{{\rm{d}}\Sigma }{{\rm{d}}\Omega }$ can be obtained by using the function


1 cs = mag2exp.sans.cross_section(field, ...)


Where the polarization of neutrons and the type of scattering observed are passed to the function. In mag2exp the different types of scattering can be written as a string and passed to the function. For example, + is denoted by p, and − is denoted by n leading to cross sections such as pn$\left(\frac{{\rm{d}}{\Sigma }^{+-}}{{\rm{d}}\Omega }\right)$, n$\left(\frac{{\rm{d}}{\Sigma }^{-}}{{\rm{d}}\Omega }\right)$, and unpol$\left(\frac{{\rm{d}}\Sigma }{{\rm{d}}\Omega }\right)$ which can be related to Eqns. (15), (16), (17), and (18). Uses of this syntax can be seen in Fig. 10.

The function calculates a 3-dimensional scattering cross section in reciprocal space. The direction of the incident beam can be set by cutting a plane normal to the beam direction through the origin. For example


1 cs.sel(k_z=0)


would be equivalent to the beam propagating along the *z* direction and


1 cs.sel(k_x=0)


would be equivalent to the beam propagating along the *x* direction. We assume that the incident beam has infinite width in real space. In combination with the polarization of the neutrons used when calculating the cross section, the common scattering geometries in SANS can be recreated.

**Fig. 9:**
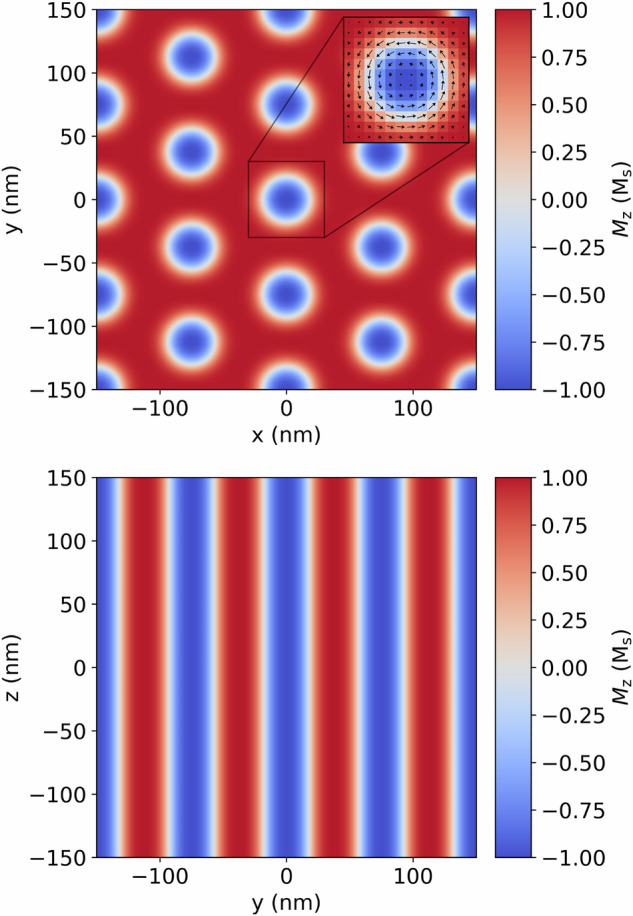
SANS magnetization. Magnetization structure used for SANS calculations. Top: *z* = 0 nm plane and Bottom: *x* = 0 nm plane.

Figure 9 shows a magnetization structure created in a 300 × 300 × 300 nm^3^ region with periodic boundary conditions and a cell size of 5 × 5 × 5 nm^3^. Deviating from the energy equation detailed in Section “Methods”, demagnetization is not included here but instead periodic boundary conditions are used to more accurately depict the scattering from a large macroscopic crystal. A skyrmion lattice is created by initializing the magnetization in a triple-q state^39^ and then allowing the system to relax and find the minimum energy configuration under the application of a 100 mT magnetic field. The system relaxes to a hexagonal skyrmion lattice with the skyrmions tubes oriented along the *z* direction of the sample.

**Fig. 10:**
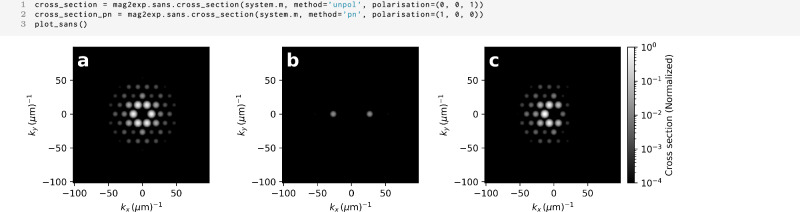
SANS cross section. Unpolarized SANS cross section for the magnetic structure shown in Fig. 9 with an incident beam along the **a** *z* direction and **b** *x* direction with initial polarization of the neutrons is in the (0, 0, 1) direction. **c** + − spin-flip SANS cross section with the incident beam along the *z* direction with the initial polarization of the neutrons in the (1, 0, 0) direction.

Figure 10(a) and (b) show the unpolarized scattering cross section for incoming neutrons polarized in the *z* direction and the beam incident in the *z* and *x* direction, respectively. These represent the two most employed scattering geometries in magnetic SANS experiments^37^. A hexagonal pattern can be seen with the incident beam along the *z* direction indicating the hexagonal symmetry of the system, whilst with the incident beam along the *x* direction three spots can be seen originating from the diffraction of the neutrons from the skyrmion tubes. This same pattern is observed in experimental measurements of skyrmions in FeGe^40^. Figure 10(c) depicts the positive to negative spin-flip SANS cross section for a beam propagating in the *z* direction with an initial polarization in the (1, 0, 0) direction. It is clearly visible that the peaks in the positive *k*_*x*_ direction have a reduced intensity, as expected, compared to the unpolarized SANS scattering cross section.

**Fig. 11:**
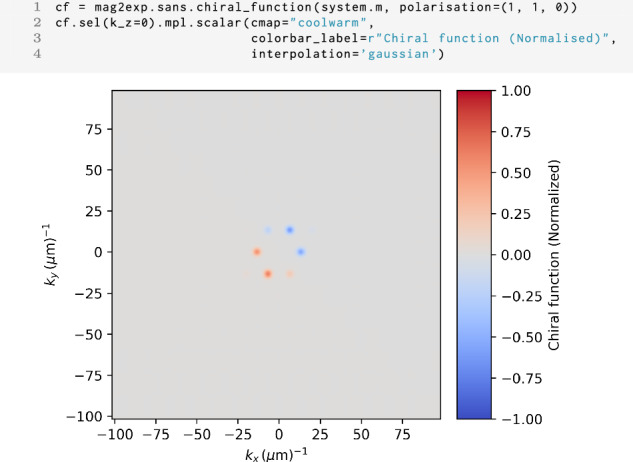
SANS chiral function. Chiral function −2*iχ* for the magnetic structure shown in Fig. 9 with the incident beam along the *z* direction. The initial polarization of the neutrons is in the (1, 1, 0) direction.


Figure 11 depicts the chiral function for a beam propagating in the *z* direction with an initial polarization in the (1, 1, 0) direction. In mag2exp, this can be calculated using

1 mag2exp.sans.chiral_function(field, ...)


Where the polarization of the incoming neutron beam can be passed. The chiral function describes spin-flip scattering and it is evident that the peaks have negative intensity in the direction of the polarization vector and positive intensity in the opposing direction.

### Ferromagnetic resonance

In addition to the static techniques described above, mag2exp has the ability to recreate the results of time-dependent measurement techniques such as Ferromagnetic resonance (FMR).

Ferromagnetic resonance is a widely used experimental method for investigating magnetization dynamics in materials. In FMR experiments, the magnetization dynamics is typically excited by an external perturbation, such as a microwave field, an applied magnetic field pulse, or a current pulse. This excitation drives the system out of equilibrium and excites resonant modes within the sample. By analyzing these modes, key material properties, such as the Gilbert damping parameter and the anisotropy fields, can be extracted^41,42^.

To compute the FMR signal from time-dependent magnetization data **M**(**r**, *t*) the local power spectrum can be determined using the spatially-resolved ringdown method

20 $${\bf{S}}({\bf{r}},f)={\left\vert \widetilde{{\bf{M}}}({\bf{r}},f)\right\vert }^{2},$$

where $\widetilde{{\bf{M}}}({\bf{r}},f)$ represents the Fourier transform of the magnetization along the time dimension. Similarly, the spatially resolved phase can be obtained by taking the argument of the Fourier transformed magnetization

21 $${\mathbf{\phi }}({\bf{r}},f)=\,{\text{Arg}}\,\left(\widetilde{{\bf{M}}}({\bf{r}},f)\right).$$

In many experimental setups, measurements typically yield only the spatially averaged power, often referred to as the Power Spectral Density (PSD). This averaged quantity can be obtained by taking the mean of Eqn. (20) over the three spatial dimensions

22 $${\bf{S}}(f)=\frac{{\int}_{V}{\bf{S}}({\bf{r}},f){{\rm{d}}}^{3}r}{{\int}_{V}{{\rm{d}}}^{3}r}\in {{\mathbb{R}}}^{3}$$

$$V=\{{\bf{r}}\,| \,{\bf{r}}\in {{\mathbb{R}}}^{3}\,\text{and}\,{\rm{valid}}({\bf{r}})={\rm{True}}\}.$$

Within the ubermag framework, time-dependent magnetization data **M**(**r**, *t*) are conveniently stored using a micromagneticdata.Drive object. The power and phase information of the FMR signal can be directly computed via the ringdown method provided by mag2exp, simply by passing the time-dependent magnetization data stored in a Drive object:


1 mag2exp.fmr.ringdown(drive, ..)


This function returns both the power and phase data as xarray.DataArray objects, which allow convenient data analysis and visualization. It can also be passed an optional argument of a discretisedfield.Field to subtract from each timestep of the drive, which allows computation of FMR on the magnetization difference.

**Fig. 12:**
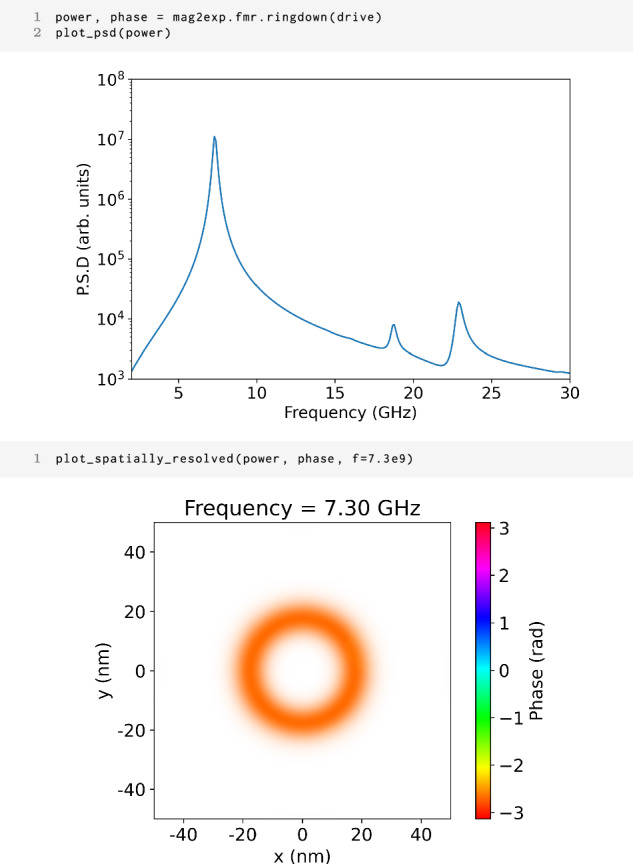
FMR spectra. Commands to calculate the power spectrum and corresponding phase information due to a perturbation of an applied magnetic field for a skyrmion in a thin film. The top image shows the power spectral density, the lower image shows the spatially resolved power and phase for the peak at 7.3 GHz, a skyrmion breathing mode. The phase is shown by the color and the opacity shows the power.

Figure 12 illustrates the spatially averaged power spectrum for an isolated skyrmion in a thin film. To obtain this spectrum, the skyrmion is driven out of equilibrium by perturbing the magnitude of the applied magnetic field, after which the system is allowed to relax. Clear resonant peaks can be seen in the power spectrum, each corresponding to a distinct resonant mode.

One major advantage of simulations is access to spatially resolved power and phase data. For example, the lower panel of Fig. 12 shows the spatial distribution of both power and phase for the resonant mode at 7.3 GHz. Here, the color encodes phase while the opacity indicates power intensity. The plot reveals that the highest power dissipation occurs uniformly around the skyrmion ring. All points on the ring have the same phase, which is characteristic of a skyrmion breathing mode.

## Discussion

The mag2exp package captures the physics of real-world experiments in a computationally readable format that allows measurements to be virtually carried out on magnetization fields. Based on the numerically or analytically obtained magnetization field, and a few technique-dependent parameters, it can simulate measurements including magnetometry, microscopy, and reciprocal space techniques, as outlined in the previous sections. The integration of mag2exp into the computational Python-based micromagnetics framework Ubermag simplifies and streamlines the typical workflows used to corroborate experimental studies and helps with reproducibility^43^. The mag2exp package has been designed to operate on arbitrary finite-difference magnetization data, such as created by computational micromagnetics packages (such as OOMMF^31^ and MuMax3^1^). However, other data sets on finite difference grids, obtained for example from Monte Carlo methods or mean-field approaches^44^ can similarly be used to provide the magnetization vector field: once the data is accessible as a numpy array it can trivially be converted to a format usable by mag2exp. We encourage contributions from the community to extend this package.

## Methods

### Virtual experiments

In order to perform the virtual experiments with mag2exp, first a magnetization structure needs to be created. This is done using a finite-difference discretization to define a mesh of cuboidal cells which contain the magnetization values. Where the geometry of interest is not a cuboid, an associated property valid which can be True or False to indicate that there is no magnetic material at that location. This enables complex simulations on systems which are heterogeneous. These restrictions are in line with the approach chosen in commonly used micromagnetic simulation packages^1,31,45^.

We use the discretisedfield package^46^, developed as part of Ubermag^3,4^, to define the magnetization at each cell on a finite-difference mesh as a discretisedfield.Field object. This package facilitates mathematical operations on finite-difference grids, such as vector operations and calculus. This enables the mag2exp functions to be expressed concisely and to be easily extensible by users. The results of the simulation of the experiment and measurement are returned as discretisedfield.Field objects. This class has convenience functions in order to analyse and plot the data, allowing efficient analysis and visualization. By re-using existing functionality we avoid duplication of effort and software.

The discretized magnetization vector field can be initialized in different ways, for example using an analytical model or by performing micromagnetic simulations. Some of the virtual experiments use this magnetization in a “read-only” fashion to passively calculate quantities while others, such as torque magnetometry, lend themselves to actively change the parameters of the micromagnetic simulations to gain more meaningful information. Additionally, if the user has experimentally obtained spatially resolved magnetization, for instance from advanced tomography^47^ it can be used with mag2exp to generate simulations of any of the experimental techniques.

All functions in mag2exp operate on a magnetization field that has to be passed as an input argument alongside relevant experimental parameters. For example, in LTEM the defocus can be passed as an input argument. Internally, the calculations are performed, and then the relevant experimental quantities are computed and returned. The different experimental techniques implemented in mag2exp are organized in multiple submodules each containing functions relevant to one experimental technique. One additional submodule, quick_plots, contains functions to each create an image that shows the outcome of the respective experimental techniques with a single line of Python code—trading ease of use against a lack of flexibility.

In mag2exp, we distinguish between two different reference frames: a sample reference frame and a technique reference frame. Both use Cartesian coordinate systems to describe the magnetization in the finite-difference mesh. The former is attached to the sample and can be arbitrarily oriented in space: the latter is attached to the experimental set-up. A few technique observables are *hard coded* into the technique reference frame. For example, MFM techniques are defined to have the magnetic tip oscillating in the *z* direction of the technique reference frame. All experiments are defined in the technique reference frame. However, rotations can be conveniently used to convert the magnetization from the sample reference frame to the technique reference frame if they are not congruent. This allows any experimental setup the user desires to be performed in the technique reference frame. Rotations of the magnetization field can be performed in the Ubermag framework using the discretisedfield package^46^.

1 field_rotator = df.FieldRotator(field)

2 field_rotator.rotate(...)

3 field_rotator.field

In this manuscript, the Object-Oriented MicroMagnetic Framework (OOMMF)^31^ was used as a computational backend to produce micromagnetic simulations in Ubermag^3–5^ allowing for realistic simulations based on real material parameters in an efficient workflow. Other computational workflows are possible, for example to feed mag2exp with data obtained using an analytical expression.

To help demonstrate selected techniques of mag2exp we set up an example micromagnetic simulation of FeGe with an additional uniaxial anisotropy. The simulation region is 300 × 300 × 20 nm^3^ with a cell size of 5 × 5 × 5 nm^3^. The energy equation consists of ferromagnetic exchange, Dzyaloshinskii-Moriya interaction (T point group), uniaxial anisotropy, Zeeman interaction, and demagnetization terms

23 $$\begin{array}{rcl}E&=&{\int}_{V}-A{\bf{m}}\cdot {\nabla }^{2}{\bf{m}}+D{\bf{m}}\cdot (\nabla \times {\bf{m}})-K{({\bf{m}}\cdot {\bf{u}})}^{2}\\ &&-{\mu }_{0}{M}_{{\rm{s}}}{\bf{m}}\cdot {\bf{H}}-\frac{1}{2}{\mu }_{0}{M}_{{\rm{s}}}{\bf{m}}\cdot {{\bf{H}}}_{{\rm{d}}},\end{array}$$

where *A* = 8.78 × 10^−12^ Jm^−1^ is the exchange constant, *D* = 1.58 × 10^−3^ Jm^−2^ is the DMI constant, *K* = 1 × 10^5^ Jm^−3^ is the uniaxial anisotropy constant, *u* = (0, 0, 1) is the uniaxial anisotropy direction, **H** an external magnetic field, *M*_s_ = 3.84 × 10^5^ Am^−1^ is the saturation magnetization, and **m** = **M**/*M*_s_ the normalized magnetization field^48^. An example of a magnetization field produced using these parameters is shown in Fig. 2. For the examples used in this paper, the magnetization field is stored in the variable system.m. It is also worth noting that the unitary Fourier transform convention with ordinary frequency is used throughout the manuscript and software.

### Research software engineering

mag2exp is an open-source Python library developed for the magnetism community. The source code is publicly available under the BSD 3-Clause License on GitHub https://github.com/ubermag/mag2expas part of the Ubermag GitHub organization. Documentation, API reference, and tutorials are available at https://ubermag.github.io. The code for these tutorials can be run in the cloud using Binder. Contributions by users and members of the community to the code, tutorials, and documentation are encouraged by creating pull requests in the mag2exp repository on GitHub. mag2exp can be installed on all major operating systems either on its own or as part of the Ubermag meta-package via pip or conda package managers.

We have chosen Python as the language because (i) there is a growing selection of Python-interfaced or Python-based scientific open source libraries available^49–52^ and we want to benefit from this global effort, (ii) a Python-based domain-specific language for micromagnetic problems^3^ and simulations exists already^4^, and (iii) Python is well supported by the Jupyter notebook, which is received by many scientists as a tool to help them analyze their data^53–55^, and also supports better reproducibility of research results^43^. As mag2exp functions are built using the discretisedfield package, users can easily use the inbuilt mathematical functions to create custom observables.

We use continuous integration using GitHub actions to execute various automatic tests after every code change^56^. These include unit, integration and system tests; tests of documentation strings; tests associated^57^ with the Jupyter-notebook based documentation. All tests are executed on Linux, OSX, and Windows operating systems.

### Supplemental material

All results obtained in this work can be reproduced from the repository in ref. ^6^ that contains software specifications and Jupyter notebooks to rerun all simulations and recreate all data and plots.

## Data Availability

The data and figures in this manuscript are available and can be fully recreated from the supplementary material https://gitlab.mpcdf.mpg.de/samholt/mag2exp_manuscript_SM.

## Code availability

The code for mag2exp is available at (https://github.com/ubermag/mag2exp, https://ubermag.github.io).

## References

[1] Vansteenkiste, A. et al. The design and verification of MuMax3. *AIP Adv.* **4**, 107133 (2014).

[2] McCray, A. R. C., Cote, T., Li, Y., Petford-Long, A. K. & Phatak, C. Understanding complex magnetic spin textures with simulation-assisted lorentz transmission electron microscopy. *Phys. Rev. Appl.* **15**, 044025 (2021).

[3] Beg, M., Pepper, R. A. & Fangohr, H. User interfaces for computational science: a domain specific language for OOMMF embedded in Python. *AIP Adv.* **7**, 056025 (2017).

[4] Beg, M., Lang, M. & Fangohr, H. Ubermag: toward more effective micromagnetic workflows. *IEEE Trans. Magn.* **58**, 1–5 (2022).

[5] Fangohr, H. et al. Vision for unified micromagnetic modeling (UMM) with Ubermag. *AIP Adv.* **14**, 015138 (2024).

[6] Supplemental Material. https://gitlab.mpcdf.mpg.de/samholt/mag2exp_manuscript_SM, 10.5281/zenodo.15097681 (2025).

[7] Porter, N., Gartside, J. C. & Marrows, C. Scattering mechanisms in textured FeGe thin films: Magnetoresistance and the anomalous Hall effect. *Phys. Rev. B* **90**, 024403 (2014).

[8] Blundell, S. *Magnetism in Condensed Matter*. Oxford master series in condensed matter physics (Oxford University Press, 2001).

[9] Perfetti, M. Cantilever torque magnetometry on coordination compounds: from theory to experiments. *Coord. Chem. Rev.* **348**, 171–186 (2017).

[10] Kumar, S. et al. Torque magnetometry study of the spin reorientation transition and temperature-dependent magnetocrystalline anisotropy in ndco5. *J. Phys. Condens. Matter* **32**, 255802 (2020). 10.1088/1361-648X/ab7ad6 32249761

[11] Prozorov, R. & Kogan, V. G. Effective demagnetizing factors of diamagnetic samples of various shapes. *Phys. Rev. Appl.* **10**, 014030 (2018).

[12] Yu, X. et al. Real-space observation of a two-dimensional skyrmion crystal. *Nature* **465**, 901–904 (2010). 20559382 10.1038/nature09124

[13] Qi, Y., Brintlinger, T. & Cumings, J. Direct observation of the ice rule in an artificial kagome spin ice. *Phys. Rev. B* **77**, 094418 (2008).

[14] Togawa, Y. et al. Magnetic soliton confinement and discretization effects arising from macroscopic coherence in a chiral spin soliton lattice. *Phys. Rev. B* **92**, 220412(R) (2015).

[15] Donnelly, C. et al. Three-dimensional magnetization structures revealed with x-ray vector nanotomography. *Nature* **547**, 328–331 (2017). 28726832 10.1038/nature23006

[16] Hirsch, P. B., Howie, A., Nicholson, R. B., Pashley, D. W. & Whelan, M. J. *Electron Microscopy of Thin Crystals* 2nd edn (Plenum Press, 1967).

[17] Loudon, J. C. et al. Do images of biskyrmions show type-II bubbles? *Adv. Mater.* **31**, 1806598 (2019). 30844122 10.1002/adma.201806598 PMC9285551

[18] Aharonov, Y. & Bohm, D. Significance of electromagnetic potentials in the quantum theory. *Phys. Rev.* **115**, 485–491 (1959).

[19] Beleggia, M. & Zhu, Y. Electron-optical phase shift of magnetic nanoparticles I. basic concepts. *Philos. Mag.* **83**, 1045–1057 (2003).

[20] Ishizuka, K. & Allman, B. Phase measurement of atomic resolution image using transport of intensity equation. *J. Electron Microsc.* **54**, 191–197 (2005). 10.1093/jmicro/dfi024 16076863

[21] Midgley, P. An introduction to off-axis electron holography. *Micron* **32**, 167–184 (2001). 10936460 10.1016/s0968-4328(99)00105-5

[22] Williams, D. B. & Carter, C. B.*Transmission Electron Microscopy—A Textbook for Materials Science* 2nd edn (Springer Science and Business Media, LLC, 2009).

[23] Kirkland, E. J. *Advanced Computing in Electron Microscopy* 2 edn. Vol 12 (Springer, 1998).

[24] Kovács, A., Li, Z.-A., Shibata, K. & Dunin-Borkowski, R. E. Lorentz microscopy and off-axis electron holography of magnetic skyrmions in FeGe. *Resolut. Discov.* **1**, 2–8 (2016).

[25] Saxton, W., Pitt, T. & Horner, M. Digital image processing: the semper system. *Ultramicroscopy* **4**, 343–353 (1979).

[26] Blume, M. & Gibbs, D. Polarization dependence of magnetic x-ray scattering. *Phys. Rev. B* **37**, 1779–1789 (1988). 10.1103/physrevb.37.1779 9944695

[27] van der Laan, G. & Figueroa, A. I. X-ray magnetic circular dichroism—a versatile tool to study magnetism. *Coord. Chem. Rev.* **277–****278**, 95–129 (2014).

[28] Turnbull, L. et al. X-ray holographic imaging of magnetic surface spirals in FeGe lamellae. *Phys. Rev. B* **106**, 064422 (2022).

[29] Kazakova, O. et al. Frontiers of magnetic force microscopy. *J. Appl. Phys.* **125**, 060901 (2019).

[30] Holt, S. J. R. *Structure and Magnetism of Skyrmion Hosting Materials*. Ph.D. thesis, University of Warwick (2021).

[31] Donahue, M. J. & Donahue, M. *OOMMF User’s Guide, Version 1.0* (US Department of Commerce, National Institute of Standards and Technology, 1999).

[32] Dussaux, A. et al. Local dynamics of topological magnetic defects in the itinerant helimagnet FeGe. *Nat. Commun.* **7**, 12430 (2016). 27535899 10.1038/ncomms12430 PMC4992142

[33] Mühlbauer, S. et al. Skyrmion lattice in a chiral magnet. *Science* **323**, 915–919 (2009). 19213914 10.1126/science.1166767

[34] Barnsley, L. C. et al. A reverse Monte Carlo algorithm to simulate two-dimensional small-angle scattering intensities. *J. Appl. Crystallogr.* **55**, 1592–1602 (2022). 36570657 10.1107/S1600576722009219 PMC9721324

[35] Michels, A. Magnetic small-angle neutron scattering of bulk ferromagnets. *J. Phys. Condens. Matter* **26**, 383201 (2014). 25180625 10.1088/0953-8984/26/38/383201

[36] Krycka, K. L. et al. Resolving 3D magnetism in nanoparticles using polarization analyzed SANS. *Phys. B Condens. Matter* **404**, 2561–2564 (2009).

[37] Mühlbauer, S. et al. Magnetic small-angle neutron scattering. *Rev. Mod. Phys.* **91**, 015004 (2019).

[38] Moon, R., Riste, T. & Koehler, W. Polarization analysis of thermal-neutron scattering. *Phys. Rev.* **181**, 920 (1969).

[39] Lancaster, T. Skyrmions in magnetic materials. *Contemp. Phys.* **60**, 246–261 (2019).

[40] Moskvin, E. et al. Complex chiral modulations in FeGe close to magnetic ordering. *Phys. Rev. Lett.* **110**, 077207 (2013). 25166404 10.1103/PhysRevLett.110.077207

[41] Baker, A. et al. Proposal of a micromagnetic standard problem for ferromagnetic resonance simulations. *J. Magn. Magn. Mater.* **421**, 428–439 (2017).

[42] Weiler, M. et al. Elastically driven ferromagnetic resonance in nickel thin films. *Phys. Rev. Lett.* **106**, 117601 (2011). 21469894 10.1103/PhysRevLett.106.117601

[43] Beg, M. et al. Using Jupyter for reproducible scientific workflows. *Comput. Sci. Eng.* **23**, 36–46 (2021).

[44] Hovorka, O. & Sluckin, T. J. A computational mean-field model of interacting non-collinear classical spins. *arXiv preprint arXiv:2007.12777* (2020).

[45] Bisotti, M.-A. et al. Fidimag–a finite difference atomistic and micromagnetic simulation package. *arXiv preprint arXiv:2002.04318* (2020).

[46] Beg, M. et al. ubermag/discretisedfield: 10.5281/zenodo.6624875 (2022).

[47] Ghidini, M. et al. Shear-strain-mediated magnetoelectric effects revealed by imaging. *Nat. Mater.* **18**, 840–845 (2019). 31110346 10.1038/s41563-019-0374-8

[48] Takagi, R. et al. Particle-size dependent structural transformation of skyrmion lattice. *Nat. Commun.* **11**, 5685 (2020). 33177528 10.1038/s41467-020-19480-8 PMC7658213

[49] Harris, C. R. et al. Array programming with NumPy. *Nature* **585**, 357–362 (2020). 32939066 10.1038/s41586-020-2649-2 PMC7759461

[50] Virtanen, P. et al. SciPy 1.0: fundamental algorithms for scientific computing in Python. *Nat. Methods* **17**, 261–272 (2020). 32015543 10.1038/s41592-019-0686-2 PMC7056644

[51] Hunter, J. D. Matplotlib: a 2D graphics environment. *Comput. Sci. Eng.* **9**, 90–95 (2007).

[52] Hoyer, S. & Hamman, J. xarray: N-D labeled arrays and datasets in Python. *J. Open Res. Softw.* **5**10.5334/jors.148 (2017).

[53] Perkel, J. M. Why Jupyter is data scientists’ computational notebook of choice. *Nature* **563**, 145–146 (2018). 30375502 10.1038/d41586-018-07196-1

[54] Fangohr, H. et al. Data exploration and analysis with Jupyter notebooks. In *Proc. 17th International Conference on Accelerator and Large Experimental Physics Control Systems (ICALEPCS'19)* (2020).

[55] Granger, B. E. & Perez, F. Jupyter: thinking and storytelling with code and data. *Comput. Sci. Eng.* **23**, 7–14 (2021).35939280

[56] Krekel, H. et al. pytest 7.1. https://github.com/pytest-dev/pytest (2004).

[57] Fangohr, H. et al. Testing with Jupyter notebooks: Notebook validation (nbval) plug-in for pytest. https://arxiv.org/abs/2001.04808 (2020).

